# Genetic Polymorphism of GSTP-1 Affects Cyclophosphamide Treatment of Autoimmune Diseases

**DOI:** 10.3390/molecules25071542

**Published:** 2020-03-28

**Authors:** Péter Hajdinák, Melinda Szabó, Emese Kiss, Lili Veress, Lívius Wunderlich, András Szarka

**Affiliations:** 1 Department of Applied Biotechnology and Food Science, Laboratory of Biochemistry and Molecular Biology, Budapest University of Technology and Economics, Szent Gellért tér 4., Budapest 1111, Hungary; livius@mail.bme.hu; 2 National Institute of Rheumatology and Physiotherapy, Clinical Immunology Adult and Pediatric Rheumatology, Frankel Leó u. 25-29., Budapest 1023, Hungarydrkiss.emese@gmail.com (E.K.); liliveress@gmail.com (L.V.); 3 Pathobiochemistry Research Group of Hungarian Academy of Sciences and Semmelweis University, P.O. Box 260, Budapest 1444, Hungary

**Keywords:** cyclophosphamide, autoimmune diseases, glutathione, glutathione-S-transferase, polymorphism

## Abstract

Cyclophosphamide is one of the most potent and reliable anti-cancer and immunosuppressive drugs. In our study, 33 individuals with different autoimmune diseases were treated with cyclophosphamide according to standard protocols. The responses to the treatments were determined by measuring the alteration of several typical parameters characterizing the given autoimmune diseases over time. We concluded that about 45% of the patients responded to the treatment. Patients were genotyped for polymorphisms of the CYP3A4, CYP2B6, GSTM1, GSTT1, and GSTP1 genes and disease remission cases were compared to the individual polymorphic genotypes. It was found that the GSTP1 I105V allelic variation significantly associated with the cyclophosphamide treatment-dependent disease-remissions. At the same time the GSH content of the erythrocytes in the patients with I105V allelic variation did not change. It appears that the individuals carrying the Ile105Val SNP in at least one copy had a significantly higher response rate to the treatment. Since this variant of GSTP1 can be characterized by lower conjugation capacity that results in an elongated and higher therapeutic dose of cyclophosphamide, our data suggest that the decreased activity of this variant of GSTP1 can be in the background of the more effective disease treatment.

## 1. Introduction

Cyclophosphamide (CYC) is a highly efficient anti-cancer drug and immunosuppressive agent. It was first synthesized in the late 1950s and became one of the best characterized and most widely administered drugs [1]. Cyclophosphamide and its derivatives are strong alkylating agents inducing the formation of monofunctional guanine-N7 adducts and interstrand guanine–guanine crosslinks inside the DNA [2]. The extensive alkylation may lead to DNA damage and consequently to the death of frequently proliferating cells.

**Figure 1:**
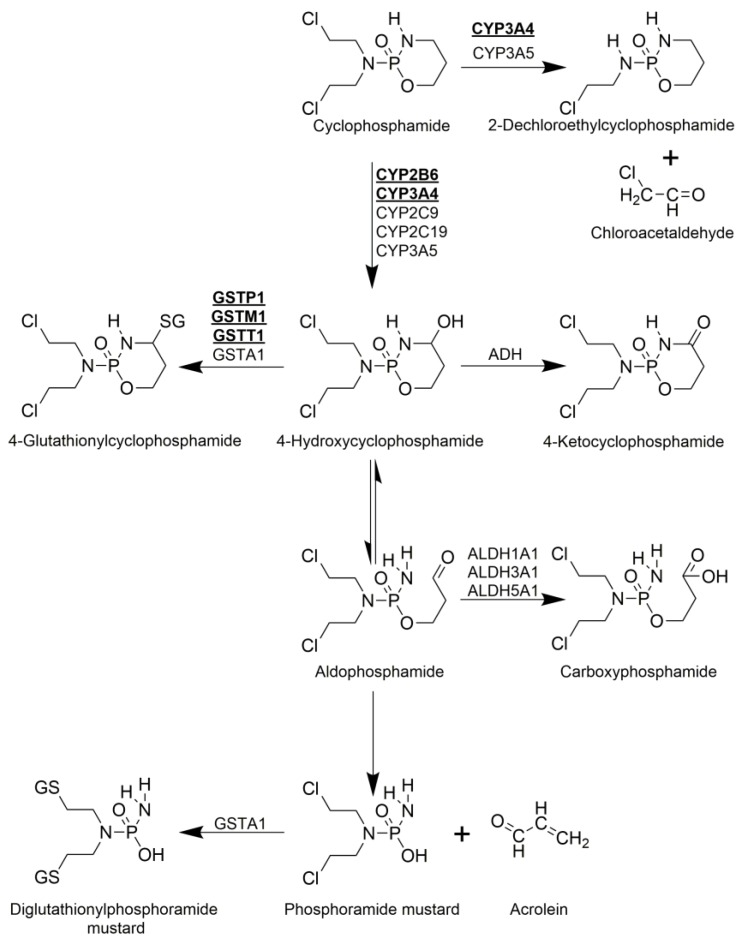
Metabolism of cyclophosphamide. Activation is shown vertically, while inactivation pathways are depicted horizontally. Cyclophosphamide (CYC) is administered as a prodrug and is hydroxylated by hepatic CYPs to form 4-hydroxycyclophosphamide (4-OH-CYC). 4-OH-CYC exists in equilibrium with its tautomer, aldophosphamide, which can break up spontaneously to result in the therapeutically active, cytotoxic phosphoramide mustard and acrolein. 4-OH-CYC can be oxidized by alcohol dehydrogenase (ADH) resulting in nontoxic 4-keto-CYC or can be conjugated with glutathione by GSTs to form 4-glutathionyl-CYC. Aldophosphamide can be oxidized by aldehyde dehydrogenases (ALDH) resulting in inactive carboxyphosphamide, while phosphoramide mustard can be detoxified by conjugating it with glutathione. Furthermore, as a minor pathway, direct detoxification of CYC is also possible by converting it to 2-Dechloroethylcyclophosphamide.

CYC is a prodrug, which is activated by biotransformation phase I enzymes. Seventy to eighty percent of the administered CYC is transformed to 4-hydroxycyclophosphamide by hepatic cytochrome P450 (CYP) enzymes (Figure 1). Several CYP isoenzymes are involved in the 4-hydroxilation of CYC in humans, including CYP3A4 and CYP2B6. The latter displays the highest 4-hydroxilase activity [3,4,5]. 4-hydroxycyclophosphamide is not cytotoxic and readily diffuses into cells [5]. Under physiological conditions, 4-hydroxycyclophosphamide exists in equilibrium with its aldehyde tautomer, aldophosphamide (Figure 1). Aldophosphamide either breaks up spontaneously into two bioactive toxic compounds such as phosphoramide mustard and acrolein or gets oxidized enzymatically by aldehyde dehydrogenases resulting in inactive, non-toxic carboxyphosphamide (Figure 1). Phosphoramide mustard, which is a bi-functional DNA alkylating agent, is the therapeutically active metabolite, while acrolein is a highly reactive aldehyde and enhances the cytotoxic effect of cyclophosphamide by depleting the cellular glutathione pool [5,6,7].

Biotransformation Phase II enzymes, such as glutathione-S-transferases (GST), catalyse the neutralization of active intermediaries of CYC by conjugation with glutathione, resulting in 4-glutathionylcyclophosphamide or diglutathionylphosphoramide mustard. The isoforms GSTM1, GSTP1, and GSTT1 are only involved in the production of 4-glutathionylcyclophosphamide (Figure 1) [4,7,8].

Although direct detoxification of CYC is also possible, less than 5% of the administered CYC is eliminated this way. The direct detoxification is catalysed by CYP3A4 and CYP3A5 and results in the formation of 2-dechloroethylcyclophosphamide via side chain oxidation [5].

The level of the bioactive phosphoramide mustard is determined by the rate of enzymatic activation and detoxification of CYC and its metabolites. The enzymes involved in CYC metabolism are known to be highly polymorphic and have alleles with decreased or missing activity [4,7]. Thus, genetic differences may be responsible for the earlier observed large interindividual variations in both efficacy and toxicity of CYC treatment [4,7,9]. For example, deletions in GSTM1, GSTT1 and some single nucleotide polymorphism of GSTP1 result in reduced detoxification rate of 4-hydroxycyclophosphamide, which may lead to a prolonged exposure to activated CYC and to the possibility of increased response. On the other hand, the prolonged exposure may increase the occurrence of adverse drug reactions [7,8]. Thus, in our study the connection between the genotype of the patients and the response to the CYC treatment in a series of chronic autoimmune diseases was investigated.

## 2. Results and Discussion

### 2.1. Patient Characteristics and Cyclophosphamide Treatment

Thirty-three patients diagnosed with various auto-immune diseases were involved in our study (Table 1). Twenty-nine of the 33 (87.9%) CYC treated patients were female and all the patients were Caucasian (Table 1). Since the vast majority of autoimmune patients are female, the female/male ratio of our study group is in concordance with the ratio in the scientific literature [10]. Their average age was 51 ± 15 years. The patients were treated one to eight times (mean ± SD: 3.03 ± 2.44) with CYC, and they received various doses of CYC occasionally (0.50 to 1.60 g, mean ± SD: 1.18 ± 0.31 g) in each pulse, 3.66 ± 3.42 g cumulative dose altogether. Upon CYC treatment, remission of the diseases was observed in 14 cases (42.42%).

### 2.2. Allele Frequencies

Allele-specific PCR discrimination method was used to genotype the study group. Homozygous wild type (WT), homozygous variant, and heterozygous allelic variations could be distinguished in the investigated (CYP2B6, CYP3A4 and GSTP1 genes) single nucleotide polymorphisms (SNPs). GSTM1 and GSTT1 deletion variants could only be detected in homozygous form. In concert with earlier studies [4,11], no variant alleles could be found in the investigated CYP3A4 SNPs (Table 2). In contrast, in the cases of CYP2B6, Q172H, and K262R wild type, heterozygous and homozygous variant patients could also be found in our study group (Table 2). The allelic frequencies of Q172H and K262R were 11% and 33% respectively. Earlier studies found similar frequencies both for K262R and Q172H [4,12,13,14]. Allelic frequencies of the investigated GST genes were quite diverse, both variant and wild type alleles were abundant in all three cases (Table 2). Their frequencies were similar to those found in Caucasians in earlier studies (Table A1) [4,7,15,16].

Alleles without genetic variability (CYP3A4 -289A->G, CYP3A4 S222P, CYP3A4 M445T) were not investigated further in our study. Regarding the polymorph alleles of the CYP2B6 (Q172H or K262R), GSTM1 and GSTT1 genes, the genetic polymorphisms could not be associated with the response to CYC treatment (Table 2). In the cases of CYP2B6, Q172H, and K262R SNPs, the heterozygous and wild type patients were compared, since there was a low number of homozygous variant patients.

The GSTP1 I105V allelic variation was associated with the CYC treatment dependent on disease remissions (Table 2). It appears that the individuals carrying the Ile105Val SNP in at least one copy had a significantly higher response rate. Since GSTP1 is active only in its homodimer form [17,18], in heterozygous patients only one fourth of the enzymes are expected to have full activity. On the basis of this consideration and the low number of patients homozygous for the variant GSTP1 allele, the groups of homozygote variant and heterozygous patients were combined and compared to the wild type, as it was also done in earlier works [7,19,20,21,22].

GSTP1 plays a major role in 4-hydoxycyclophosphamide conjugation with about 1.9 mM of K_M_ and 35.1 nmol/min/mg protein of V_max_ values [8]. The latter high value underlines the importance of GSTP1 in CYC detoxification. Since the variant isoform of GSTP1 can be characterized by lower conjugation capacity [23,24,25] that results in an elongated and higher therapeutic dose of CYC, our data suggest that the decreased activity of the variant GSTP1 can be in the background of the higher response rate. Necessarily, the limited number of patients we could involve in the study and the facts that they were all diagnosed with autoimmune diseases and most of them were women might distort the allele frequencies and our results. However, our findings are in concert with earlier works. Several studies reported association between GSTP1 I105V polymorphism and increased response to cyclophosphamide-based therapies in breast and other cancer patients [24,26]. The effects of the variant allele included improved overall survival and decreased rate of relapse for both heterozygous and homozygous variant patients [24,26,27,28,29,30]. Due to its lower detoxification capacity, the I105V polymorphism may also have negative effects. Zhong et al. [31] reported increased risk of myelotoxicity and gastrointestinal toxicity in patients with systemic lupus erythematosus carrying at least one copy of the variant allele when they are treated with CYC. Another study reported that, carrying at least one allele of I105V results in a significantly increased risk of developing therapy-related acute myeloid leukemia after cytotoxic chemotherapy [32]. At the same time, there are studies that did not find any effect of I105V polymorphism on the CYC therapy, or the polymorphism showed a nonsignificant trend toward a lower probability of achieving global remission [4,7]. It seems that ethnicity differences and study group sizes also influence the results regarding the effect of GSTP1 I105V [26].

### 2.3. Blood Glutathione Levels

Blood samples were taken from each patient right before and 8 h after the first CYC treatment then before and after the CYC treatment when its dose was adjusted to determine the possible glutathione (GSH) level fluctuations. The latter is presented in the manuscript, but significant differences could not be observed between the GSH levels of the two samples (data not shown). The GSH level of both the plasma and the erythrocytes was measured. As it was expected on the base of the earlier observations [33,34,35], the GSH concentration in the erythrocytes was much higher than in the plasma (Table 3). No significant changes in GSH levels from both sources was found due to CYC treatment by using Wilcoxon matched pairs test (Table 3).

It has been found earlier, that almost exclusively the P1 isoform of GST can be found in the erythrocytes [36]. Since GSH levels were high enough in most of the erythrocyte samples, it is expected that GSH was not a limiting factor for the conjugation. In general, 2200 μmol/(L red blood cells) of GSH concentration could be measured in the erythrocytes, and the K_M_ for GSH of the human erythrocyte GST was around 110 µM [37]. It should be noted that there is no measurable GST activity in the plasma, and the erythrocytes are considered to be the main carriers of 4-hydroxycylophosphamide [5,37,38,39,40]. On the basis of these considerations, we have compared the erythrocyte GSH levels of patients carrying the variant GSTP1 I105V allele in at least one copy to the wild type patients (Table 3). We found no significant differences between these groups, which suggests that this polymorphism does not affect the erythrocyte GSH levels. Thus, our results for erythrocyte glutathione content further support our hypothesis, that the decreased catalytic activity of the variant GSTP1 enzyme (both heterozygous and homozygous variant) can be responsible for the increased response rate to therapeutic CYC.

### 2.4. Other Parameters

Further characteristic parameters of the patients responding and not responding to CYC treatment were compared (Table 4). Blood samples taken a couple days before CYC treatment were investigated. While there were no significant differences, some tendencies could be found. The samples from responding individuals could be characterized by lower erythrocyte sedimentation rates and lower C-reactive protein levels than those from non-responding patients. Interestingly, we also found some non-significant trends (*p* = 0.10) indicating that lower dosage cyclophosphamide treatment might have resulted in slightly higher disease remission rates. These phenomena should be investigated in a larger population. We have also compared the ages of the successfully and unsuccessfully treated groups, but no significant difference has been found.

## 3. Patients and Methods

### 3.1. Cyclophosphamide Treatment

Patients diagnosed with large vessel vasculitis (LVV), antineutrophil cytoplasmic antibody associated vasculitis (AAV), dermatomyositis (DM), systemic sclerosis (SSc), systemic lupus erythematosus (SLE), retroperitoneal fibrosis (RF), interstitial lung disease (ILD) or sarcoidosis and treated with CYC were chosen to take part in our study. The ethnicity of all patients was Caucasian.

CYC treatments were carried out according to the standard medical protocols of the given diseases. A patient was defined as a responder if remission of the symptoms was clearly observed and further biological therapies were not necessary to apply. In the case of the response, the treatment was continued by per *os* administration of synthetic disease-modifying antirheumatic drugs (DMARDs).

This survey was conducted in compliance with the protocol of Good Clinical Practices and Declaration of Helsinki principles and was carried out with the approval of the Ethics Committee of the Medical Research Council, Research and Ethics Committee (TUKEB; GLUTCYC-1-2016). All participants gave a written informed consent.

### 3.2. DNA Sample Preparation

Buccal cells were collected by rubbing the inner surface of the cheek’s epithelium with sterile cotton swab sticks for about 30 s. The buns were separated by cutting the stick with scissors and placed in sterile microcentrifuge tubes containing 0.5 mL lysis buffer (0.1 M NaCl, 0.01M Tris-HCl, 0.5% SDS, 0.2 mg/mL proteinase K, pH 8). The collected samples were incubated for 12 h at 56 °C, and the cotton swabs were removed after the incubation period. One-hundred-and-sixty-seven microliters of 6 M NaCl solution was added to the lysates, followed by centrifugation at 17,000× *g*, for 10 min (room temperature). The supernatants were transferred into new tubes and supplemented with 690 μL of chilled (−20 °C) isopropyl alcohol. After an incubation at −20 °C for 2 h, the samples were centrifuged at 20,000× *g,* 4 °C for 20 min and the supernatants were carefully removed. The DNA pellets were washed with −20 °C 70% (*v*/*v*) ethanol, and centrifuged (20,000× *g*, 4 °C, 20 min) again. After removal of the supernatants, the pellets were dried and dissolved in 50 μL TE buffer (5 mM Tris, 0.5 mM EDTA, pH 8). The DNA concentrations were measured with Nanodrop Spectrophotometer (Thermo Fisher Scientific, Waltham, MA, USA) and the samples were stored at −80 °C until use.

### 3.3. PCR

The analysis of CYP and GST polymorphisms was carried out by a standard, three-step polymerase chain reactions (PCR) followed by agarose gel electrophoresis. Allele variations of CYP3A4 (CYP3A4*1B (-289A->G), CYP3A4*2 (S222P), CYP3A4*3 (M445T)), CYP2B6 (Q172H & K262R), and GSTP1 (I105V) were investigated. GSTM1 and GSTT1 null mutations were also analysed. The nucleotide sequences of the primers and the used annealing temperatures are listed in Table A2.

### 3.4. Determination of Blood Glutathione Content

Venous blood samples were collected into EDTA-VACUETTE tubes (Greiner, Kremsmünster, Austria) right before and 8 h after CYC treatment. Blood samples were immediately centrifuged for separating the erythrocyte and plasma fractions (at 3000× *g* for 5 min, at room temperature). 0.5 mL 10% (*w*/*v*) 5-sulphosalycilic acid (SSA, Sigma-Aldrich, St. Louis, MO, USA) solutions were mixed to the same volume of both fractions. The samples were stored at −80 °C for no more than 2 days.

Before the derivatization of the glutathione (GSH) content of the samples with the fluorescent dye monochlorobimane, the frozen samples were thawed, centrifuged (17000× *g*, 5 min, room temperature) and 100 μL of the supernatants were transferred into new tubes. Triethanolamine was added to the samples at a final concentration of 400 mM to adjust the pH to being slightly alkaline. The GSH content of the samples was conjugated immediately by monochlorobimane (1 mM) in the presence of glutathione-S-transferase enzyme (100 mU/mL). The reaction was carried out in the dark at room temperature for 15 min and stopped with the addition of trichloroacetic acid at a final concentration of 10% (*w*/*v*) [41,42]. The samples were centrifuged at 16,000× *g*, at 4 °C for 10 min to pellet the precipitated proteins. Fifty microliters of the supernatant was loaded into a Waters 2690 HPLC for separation equipped with a Waters 2475 fluorescent detector set to 395 nm excitation and 477 nm emission wavelengths. Samples and standards were separated on a Teknokroma Nucleosil 100C-18 (Teknokroma Analítica, Barcelona, Spain), 5 μm, 250 × 4.6 mm column. The separation was carried out with a 32.5 min protocol employing the linear gradient of 0.25% (*v*/*v*) aqueous acetic acid/sodium acetate buffer pH 3.5 as Solvent A and methanol as Solvent B: 0–17.5 min 18% Solvent B/ 82% Solvent A; 17.5–20 min linear gradient to 100% Solvent B; 20–27.5 min Solvent B; 27.5–28 min linear gradient to 18% Solvent B/ 82% Solvent A; 28 to 32.5 min 18% Solvent B/ 82% Solvent A with 1 mL/min permanent flow rate. Standards were prepared from reduced glutathione dissolved in 5% SSA [0–20 µM], and their glutathione content (similarly to the samples) was conjugated with monochlorobimane. Based on our standards, the retention time of glutathione conjugated by monochorobimane was 8.5 min [42].

### 3.5. Statistical Analysis

For the primary data analysis, Chi-square test was used to compare response rates between genotype groups (categorical variables). If the observed frequency in a cell of the contingency table was <5, then Fisher’s exact test was used. On reviewing our results, we conducted a post-hoc analysis assuming a dominant genetic effect in the case of GSTP1 I105V [7,19,20,21,22]. Therefore, heterozygous and homozygous variant patients were combined and compared to wild type patients using Fisher’s exact test. The *p*-value presented for GSTP1 I105V in Table 2 came from this post-hoc analysis.

The non-parametric Mann-Whitney and Wilcoxon matched pairs tests were used to compare continuous variables. Mann-Whitney test was used to analyse the erythrocyte GSH with GSTP1 genotype as grouping variable; age; erythrocyte sedimentation rate; C-reactive protein and cumulative dose of CYC. For comparing blood glutathione levels before and 8 h after CYC treatment, Wilcoxon matched pairs test was used. Statistical analyses were performed by StatSoft STATISTICA^TM^ 10 (StatSoft, Tulsa, OK, USA, 2010).

## 4. Conclusions

CYC treatment is one of the most common and reliable possibilities for suppressing the symptoms of autoimmune diseases. Unfortunately, high interindividual variations in treatment efficacy and response have been observed. One of the factors influencing the response to the treatment can be the genetic background of the patients. In our study, we focused on the bioactivation and elimination of CYC. The relations of the CYC treatment response and various biotransformation-dependent factors such as CYP and GST enzyme polymorphisms and GSH levels of the blood were investigated.

On one hand, a clear association between GSTP1 genetic polymorphism and CYC treatment response was found. Significantly higher response rate to CYC treatment could be observed in individuals carrying one or two copies of the gene of the less active variant GSTP1 (I105V) isoenzyme. Since GSTP1 is the main GST isotype in the erythrocytes, it is not surprising that the weakened first detoxification reaction may result in a higher amount of 4-hydroxycyclophospahmide transported into the proliferating cells, that can enhance its cytostatic effect.

On the other hand, no significant blood GSH depletion could be observed upon CYC treatment. This observation suggests that GSH level was not a limiting factor for the biotransformation. According to these findings, it seems that CYC treatment has little effect on the glutathione pool in the erythrocytes.

## Figures and Tables

**Table 1:** Patient characteristics at baseline and outcome of the CYC treatment. Thirty-three patients diagnosed with various autoimmune diseases were included in the study. Data in square brackets represent the range of the given trait in the study population.

Clinical characteristics (n = 33)	
Sex, n	29 female/ 4 male
Ethnic origin, Caucasian, [%] (n)	100.00 (33)
Age at sample collection, mean ± SD	50.81 ± 15.24 [24–82]
**Treatment**	
Cumulative dose of CYC, mean ± SD [g]	3.66 ± 3.42 [0.5–12.6]
CYC pulses, mean ± SD	3.03 ± 2.44 [1–8]
Response to CYC treatment, [%] (n)	42.42% (14)
**Biological characteristics**	
Erythrocyte sedimentation rate, mean ± SD [mm/h]	26.84 ± 17.17 [4–67]
C-reactive protein, mean ± SD [mg/L]	8.43 ± 7.14 [1.15–34.12]
**Diseases**	
ANCA-associated vasculitis, [%] (n)	18.18 (6)
Large vessel vasculitis, [%] (n)	3.03 (1)
Systemic sclerosis, [%] (n)	27.27 (9)
Interstitial lung disease, [%] (n)	12.12 (4)
Systemic lupus erythematosus, [%] (n)	21.21 (7)
Retroperitoneal fibrosis, [%] (n)	6.06 (2)
Dermatomyositis, [%] (n)	6.06 (2)
Sarcoidosis, [%] (n)	6.06 (2)

**Table 2:** Association between response to CYC treatment and CYP3A4, CYP2B6, GSTM1, GSTP1, and GSTT1 polymorphisms. Significant association was only found between GSTP1 (I105V) variant and response to CYC treatment (*p* < 0.05).

.	WT Responders/All Carriers [n], (%)	Heterozygous Variant Responders/All Carriers [n], (%)	Homozygous Variant Responders/All Carriers [n], (%)	*p*
CYP3A4*2 (S222P)	14/33 (42.42%)	—	—	N/A
CYP3A4*1B (-289A->G)	14/33 (42.42%)	—	—	N/A
CYP3A4*3 (M445T)	14/33 (42.42%)	—	—	N/A
**CYP2B6 (Q172H)**	12/27 (44.44%)	2/5 (40%)	0/1 (0%)	0.62
**CYP2B6 (K262R)**	4/14 (28.57%)	9/16 (56.25%)	1/3 (33%)	0.12
**GSTM1 (deletion)**	7/16 (43.75%)	N/A ^1^	7/17 (41.17%)	0.88
**GSTP1 (I105V)**	3/14 (21.42%)	8/13 (61.53%)	3/6 (50%)	0.03*
**GSTT1 (deletion)**	9/24 (37.5%)	N/A ^1^	4/8 (50%)	0.41

^1^ GSTM1 and GSTT1 deletion variants could only be detected in homozygous form. * The presented p value is from a post-hoc analysis assuming a dominant genetic model.

**Table 3:** Plasma and erythrocyte glutathione content of 33 patients before and 8 h after cyclophosphamide treatment. No significant changes in glutathione levels have been observed.

		All Patients	*p* *	WT for GSTP1	GSTP1 I105V Carriers	*p* **
**Plasma GSH content before treatment, mean ± SD**	[µM]	0.47 ± 0.56	0.68	**—**	**—**	**—**
**Plasma GSH content 8 h after treatment, mean ± SD**	[µM]	0.64 ± 0.82		**—**	**—**	**—**
**Erythrocyte GSH content before treatment, mean ± SD**	[µmol/L red blood cells]	2285.50 ± 1822.34	0.73	2172.56 ± 1519.79	2271.03 ± 2069.04	0.90
**Erythrocyte GSH content 8 h after treatment, mean ± SD**	[µmol/L red blood cells]	2472.80 ± 2235.55		2334.50 ± 2076.64	2853.45 ± 2421.49	1.00

* The *p*-value presented is from comparing the plasma and erythrocyte GSH contents of all patients before and 8 h after CYC treatment. ** The *p*-value presented is from comparing the erythrocyte GSH content of WT and GSTP1 I105V carrier patients before and 8 h after CYC treatment.

**Table 4:** Comparison of variables of patients responding and not responding to CYC treatment. The data of 33 patients were analyzed. No significant differences between the responding and non-responding populations were observed.

	Responder, Mean ± SD	Non-responder, Mean ± SD	*p*
**Age**	49.21 ± 19.06	52.05 ± 11.93	0.54
**Erythrocyte sedimentation rate** **, [mm/h]**	21.28 ± 13.28	31.16 ± 18.90	0.15
**C-reactive protein, [mg/L]**	5.05 ± 3.34	10.70 ± 8.48	0.08
**Cumulative dose of CYC [g]**	2.87 ± 3.12	4.47 ± 3.57	0.10

## Appendix A

**Table A1:** Allele frequency of GSTP1 I105V in various populations.

Study Population Ancestry	GSTP1 I105V Frequency [%]	Ref.
Tanzanian	16	[43]
South African Venda	12	
Zimbabwean	21	
Gambian	53	[44]
Dutch	53	[4]
French	46	[7]
Serbian	61 *	[45]
Turkish	29 **	[46]
Han Chinese	20.5	[31]
Bangladeshi	29	[26]
Delhi	32	[47]

* % of the study group carrying at least on copy of the variant allele; ** % of the studied haplotypes has I105V.

**Table A2:** Primers, annealing temperatures and polymerization length used for genotyping.

Genetic Variations	Reverse and Forward Primers (5′-3′)	Ann. Temp. (°C)	Polymerization Length (sec)	Product Length
**CYP3A4*1B (-289A->G)**	GGAAACAGGCGTGGAAACAC and CCTTTGAGTTCATATTCTATGAGGTATGAT or CCTTTGAGTTCATATTCTATGAGGTATGAC	59	30	416 bp
**CYP3A4*2 (S222P)**	TTTGATTTTTTGGATCCATTCTTTCACT or TTTGATTTTTTGGATCCATTCTTTCACC and CAATGCCCTAATCTCTTTGCCT	58	60	867 bp
**CYP3A4*3 (M445T)**	ACCCAGAAACTGCATTGGTAT or ACCCAGAAACTGCATTGGTAC and TGGGCCTAATTGATTCTTTGGC	60	20	237 bp
**CYP2B6 (Q172H)**	CAGTGCTGAGCCTGGTGTAT and ATGATGTTGGCGGTAATGCAC or ATGATGTTGGCGGTAATGCAA	54	60	931 bp
**CYP2B6 (K262R)**	GGCGGTCTGATCTGGAAAGT and GGTAGGTGTCGATGAGGTTCT or GGTAGGTGTCGATGAGGTTCC	60	60	859 bp
**GSTP1 (I105V)**	GGACCTCCGCTGCAAATCCA or GGACCTCCGCTGCAAATCCG and ACATAGTCATCCTTGCCCGC	60	60	928 bp
**GSTM1 del**	GAACTCCCTGAAAAGCTAAAGC and GTTGGGCTCAAATATACGGTGG	57	20	219 bp [48]
**GSTT1 del**	TTCCTTACTGGTCCTCACATCTC and TCACCGGATCATGGCCAGCA	60.8	30	480 bp [49]
